# Analgesic-antitumor peptide induces apoptosis and inhibits the proliferation of SW480 human colon cancer cells

**DOI:** 10.3892/ol.2012.1049

**Published:** 2012-11-27

**Authors:** YU GU, SHEN-LIN LIU, WEN-ZHENG JU, CHANG-YIN LI, PENG CAO

**Affiliations:** 1 The First Clinical Medical College, Nanjing University of Traditional Medicine; Nanjing, Jiangsu, P.R. China; 2 Laboratory of Clinical Pharmacokinetics of Traditional Chinese Medicine, Jiangsu Province Hospital of Traditional Chinese Medicine; Nanjing, Jiangsu, P.R. China; 3 Laboratory of Cellular and Molecular Biology, Jiangsu Province Institute of Traditional Chinese Medicine, Nanjing, Jiangsu, P.R. China

**Keywords:** analgesic-antitumor peptide, colorectal cancer, apoptosis, cell cycle, proliferation

## Abstract

Colorectal cancer is one of the most common malignant tumors, and is associated with significant morbidity and mortality. In this study, recombinant analgesic-antitumor peptide (rAGAP), a protein consisting of small ubiquitin-related modifier (SUMO) linked with a hexa-histidine tag, was used as an antitumor analgesic peptide. The purpose of the present study was to investigate the antitumor activity of rAGAP in human colon adenocarcinoma SW480 cells and its potential molecular mechanisms of action. In this study, cell viability and apoptosis of rAGAP-treated SW480 cells was evaluated by the 3-(4,5-dimethylthiazol-2-yl)-2,5-diphenyltetrazolium bromide (MTT) assay, flow cytometry and 4′,6-diamidino-2-phenylindole (DAPI) staining. Western blotting was used to investigate the effects of rAGAP on p27, Bcl-2/Bax and PTEN/PI3K/Akt cellular signal transduction. Our results showed that rAGAP not only enhanced apoptosis, but also inhibited the proliferation of SW480 cells. rAGAP upregulates the expression of p27 in SW480 cells and leads to cell cycle arrest in the G1 phase. Furthermore, rAGAP significantly increases the production of Bax and PTEN and suppresses the activation of Bcl-2, phosphatidylinositol 3-kinase (PI3K) and phospho-Akt (p-Akt) in SW480 cells. These results suggest that rAGAP may be a potential new anti-colorectal cancer drug.

## Introduction

Colorectal cancer (CRC) is a common malignant tumor with relatively poor prognosis. The highest incidence rates are found in Australia, New Zealand, Europe and North America, whereas the lowest rates are found in Africa and South-Central Asia. However, CRC incidence rates are rapidly increasing in several areas historically at low risk, including Spain, and a number of countries within Eastern Asia and Eastern Europe (1). Approximately 608,000 mortalities from CRC are estimated to occur every year worldwide, accounting for 8% of all cancer-related mortalities, making it the fourth most common cause of death from cancer (2). Chemotherapy for CRC consists of irinotecan, oxaliplatin, leucovorin and 5-fluorouracil (3), providing a better prognosis for patients with advanced CRC. However, its clinical application is greatly limited by the resistance to chemotherapy and toxic side-effects. Therefore, an urgent need exists to find new strategies and drugs for CRC therapy.

Over the past years, owing to its remarkable anticancer effect and minor toxicity, traditional Chinese medicine has been of ever-increasing interest for cancer therapy. Oriental Asiatic scorpion (OAS), which originates from the dried body of *Buthus martensii* Karsch, family Buthidae, has been traditionally used in China for stroke, epilepsy, spasm, migraine and tetanus, as well as tumors, for almost 2,000 years. Various toxic polypeptides, usually called scorpion toxins, were considered to be the major bioactive ingredients of OAS. Recently, one of these polypeptides, analgesic-antitumor peptide (AGAP), has been purified and its analgesic and antitumor activities have also been demonstrated in pharmacological studies (4,5). Small ubiquitin-related modifier-AGAP (SUMO-AGAP) is a product of recombinant AGAP (rAGAP) linked with a hexa-histidine tag by *Escherichia coli* (*E. coli*). In this expression system, SUMO was capable of significantly enhancing the expression and efficiently improving the accurate folding of AGAP; thereby, rAGAP significantly inhibited the proliferation of lymphoma and glioma (5). Accordingly, rAGAP appears to be an ideal candidate drug for antitumor therapy. However, so far no study has been published on the antitumor effects of rAGAP on CRC. It is well known that cell cycle arrest and apoptosis inhibition are two major action mechanisms of antitumor drugs. Recent studies revealed that both the p27 and PTEN/PI3K/Akt pathways played important roles in cell cycle progression, while Bcl-2 family proteins regulated mitochondrial membrane permeability and played an important role in the mitochondrial apoptosis pathway (6).

In the present study, the effects of rAGAP on viability, proliferation and apoptosis of SW480 colon cancer cells were analyzed to demonstrate its antitumor bioactivity in CRC. Following this, the roles of rAGAP on protein expression of p27 and Bcl-2/Bax, as well as regulation of PTEN/PI3K/Akt signal transduction, were further investigated to illustrate the potential molecular mechanism.

## Materials and methods

### Preparation of rAGAP

rAGAP was obtained by the expression of pET28a/SUMO-AGAP in *E. coli* as previously described (5). The activity of rAGAP was the same as described previously (5). The lyophilized rAGAP powder was dissolved in phosphate-buffered saline (PBS).

### Cell culture, reagents and antibodies

The human colonic adenocarcinoma cell line SW480 was obtained from the Type Culture collection of the Chinese Academy of Sciences (Shanghai, China). When SW480 cells grew to ∼80% of the whole flask, the medium was removed and washed with PBS 3 times. Trypsin (0.25%; Invitrogen Life Technologies, Carlsbad, CA, USA) solution was added into the flask and incubated at 37°C for 1–2 min. Then 4 ml DMEM (containing 10% FBS; Invitrogen Life Technologies) was added into the flask to terminate digestion. After being centrifuged for 5 min at a speed of 1,000 rpm, the medium was replaced by fresh medium and cell suspension was divided into 2–3 culture flasks. The flasks were incubated in a humidified atmosphere of 5% CO_2_ at 37°C for 24 h. MTT and DAPI reagents were employed (Sigma, St. Louis, MO, USA) as well as antibodies against p27, Bcl-2, Bax, PTEN, PI3K, Akt, p-Akt and β-actin (all purchased from Santa Cruz Biotechnology, Inc., Santa Cruz, CA, USA).

The study was approved by the Ethics Committee of Nanjing University of Traditional Medicine (Nanjing, Jiangsu, China).

### MTT assays

SW480 cells were seeded in 96-well plastic plates (Nunc, Roskilde, Denmark) at a density of 1×10^4^ cells/well and incubated in a humidified atmosphere of 5% CO_2_ at 37°C for 24 h. After treatment with various concentrations of rAGAP (0, 5, 10, 20, 40 and 80 *μ*M) for 24 h, cell viability assays were performed using the MTT method. DMEM was added in the control group. Then, 5 mg/ml MTT solution (20 *μ*l/well) was added to each well and cells were incubated for an additional 4 h at 37°C. The medium was carefully removed and formazan crystals were dissolved in 150 *μ*l DMSO per well, and the absorbance at 490 nm was measured on a microplate reader (Bio-Rad Laboratories, Hercules, CA, USA) with DMSO as a blank control. All MTT assays were conducted independently and in triplicate. The absorbance values were presented as relative viable cell number. The growth inhibition was calculated according to the following formula: Growth inhibition rate (IR%) = (1 − absorbance values of treated samples / absorbance values of untreated samples) × 100.

### DAPI staining assay

SW480 cells (1×10^4^ per well) were seeded in six-well plastic plates and incubated for 24 h. Then various concentrations of rAGAP (0, 5, 10 and 20 *μ*M) were directly added to the well and incubated for an additional 24 h. Cells were washed briefly with cold PBS. A quantity of 0.5 ml 4% paraformaldehyde was added to each well and fixed on ice for 20 min. The supernatant was aspirated and cells were washed twice with PBS. Sodium citrate (0.1%) containing 0.1% Triton X-100 was added to each well and cells were incubated for 2 min at 4°C. Then, DAPI was added to each well after washing with PBS again. The final concentration was 1 *μ*g/ml and cells were incubated for 10 min on ice in the dark. The supernatant was aspirated and cells were washed twice with PBS. Apoptotic cells were excitated by Ultraviolet (UV). Then cell morphology was observed and photographed under fluorescence microscopy (×400; Zeiss Axio Observer A1) at 340 nm.

### Cell cycle analysis

SW480 cells were seeded onto six-well plates at a density of 1×10^6^ cells/well and incubated for one day. Following treatment with various concentrations of rAGAP (0, 5, 10 and 20 *μ*M) for 24 h, the cells were collected and washed with 1X PBS. Cell pellets were fixed in 70% cold ethanol overnight at 4°C. The fixed cells were then resuspended in 1X PBS containing 1 mg/ml RNase A, incubated for 1 h at 37°C and the cells were stained by adding 50 *μ*g/ml DNA-binding dye propidium iodide (PI) for 30 min at room temperature in the dark. The DNA contents of the stained cells were analyzed using CellQuest Software with a FACS Vantage SE flow cytometer (Becton Dickinson, Heidelberg, Germany). For each sample, a minimum of 10^4^ events were recorded.

### Western blot analysis

SW480 cells were seeded into six-well plates at a density of 1×10^6^ cells/well for protein extraction. After SW480 cells were treated with various concentrations of rAGAP (0, 5, 10 and 20 *μ*M), the cells were lysed with ice-cold RIPA buffer containing 15 mM Tris-HCl (pH 8.8), H_2_O, 30% acrylamide, 10% SDS, 10% ammonium persulfate (AP) and N,N,N′,N′-tetramethylethylenediamine (TEMED; Biosharp, Seattle, WA, USA) to obtain the total cell lysates. The total cell lysates were then centrifuged at 15,000 rpm for 15 min at 4°C to remove the insoluble materials. Next, the protein concentrations were determined using a BCA protein assay kit (Thermo Scientific, Rockford, IL, USA). Protein extracts (50 *μ*g) were analyzed using 12% polyacrylamide gel electrophoresis and electrotransferred to nitrocellulose membranes at 150 mA for 1 h. The polyvinylidene fluoride (PVDF) membranes (Millipore Corporation, Bedford, MA, USA) were then blocked for 2 h at room temperature with PBS containing 5% skimmed milk and 0.1% Tween-20 and incubated with 1:1,000 dilutions of primary antibodies including anti-p27 (1:500), anti-Bcl-2 (1:500), anti-Bax (1:500), anti-PTEN (1:200), anti-PI3K (1:500), anti-Akt (1:500) and anti-p-Akt (1:200) overnight at 4°C and subsequently with a 1:10,000 dilution of horseradish peroxidase-conjugated anti-rabbit secondary antibodies (Bioworlde, Technology, Minneapolis, MN, USA) for 1 h at room temperature. Peroxidase activity was visualized using the ECL kit (Thermo). Anti-β-actin (Santa Cruz Biotechnology, Inc.) was used as a loading control for total lysates and nuclear extracts. Immunoreactive protein bands were detected with GelDoc 2000 System (Bio-Rad). The relative protein content was represented through the gray value ratio of protein bands/β-actin protein bands, and the results were analyzed with Quantity One software.

### Statistical analysis

All data were expressed as the mean ± SD from at least three independent experiments. One-way ANOVA was used for all statistical comparisons, and the LSD t-test was conducted for multiple comparisons. P<0.05 was considered to indicate a statistically significant result. All analyses were performed using SPSS ver. 14 (SPSS, Chicago, IL, USA).

## Results

### rAGAP inhibited cell viability of SW480

**Figure 1.**
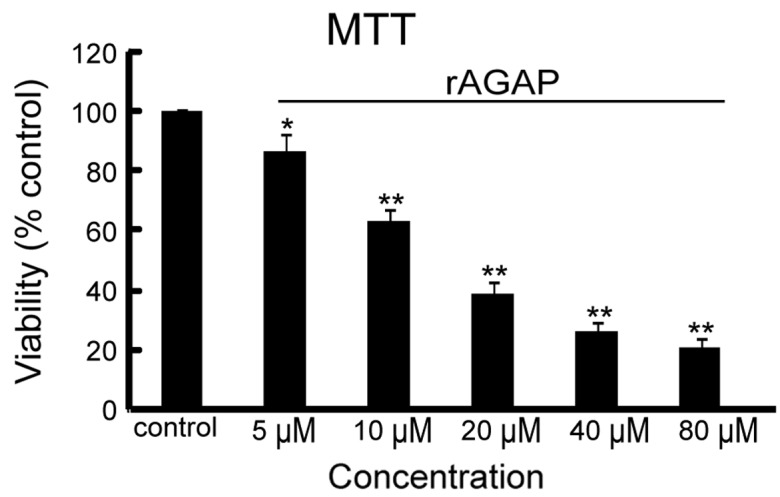
Inhibition of rAGAP on SW480 cell viability. Cell viability was measured by MTT following rAGAP treatment for 24 h. Each value was expressed as mean ± SD (n=3). ^*^P<0.05 vs. control. ^**^P<0.01 vs. control. rAGAP, recombinant analgesic-antitumor peptide.

To assess the effect of rAGAP on cell viability, different concentrations (5, 10, 20, 40, 60, 80 and 100 *μ*M) of rAGAP were incubated with SW480 cells for 24 h followed by MTT assay. As shown in Fig. 1, rAGAP inhibited the viability of SW480 cells in a dose-dependent manner. Compared with the control group, the viability of SW480 cells decreased from 87 to 21.2% at concentrations of 5, 10, 20, 40 and 80 *μ*M of rAGAP in a dose-dependent manner (IC_50_=18.4 *μ*M).

### Effect of rAGAP on SW480 apoptosis

**Figure 2.**
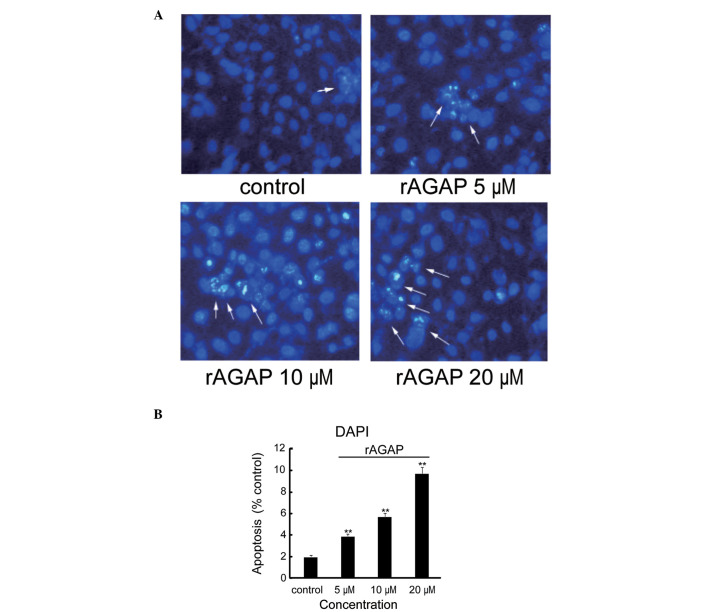
SW480 cell morphology with DAPI staining. (A) Representative images of SW480 cells treated with 0, 5, 10 and 20 *μ*M rAGAP, respectively. (B) rAGAP enhanced apoptosis of SW480 cellz. The percentage of apoptotic cells was determined from randomly selected fields of view. Statistical analysis of cell apoptosis ratio compared with control. ^**^P<0.01 vs. control, n=3. rAGAP, recombinant analgesic-antitumor peptide.

DAPI staining was used to investigate the effects of rAGAP on SW480 cell apoptosis. As shown in Fig. 2, the DAPI dye stained morphologically normal nuclei blue. Nuclear morphology of the cells in the control group was round, sharp edged and uniformly stained. Apoptotic cells were characterized by shrunken and fragmented nuclei with condensed chromatin. The ratio of apoptotic cells was 2.0, 3.9, 5.7 and 9.8% in the control, 5, 10 and 20 *μ*M rAGAP groups, respectively [F(3,8)=276.69, P<0.01].

### Effects of rAGAP on cell cycle of SW480 cells

**Figure 3.**
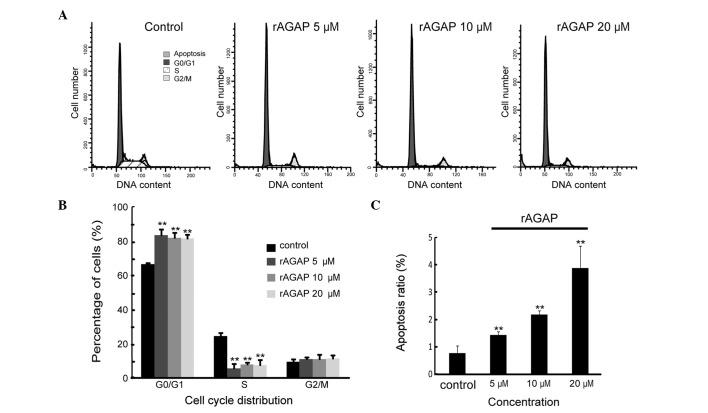
Effects of rAGAP on the cell cycle of SW480 cells. Cells were treated with rAGAP for 24 h. (A) and (B) Cell cycle distributions. (C) Apoptosis rate of cells. ^**^P<0.01 vs. control, n=3. rAGAP, recombinant analgesic-antitumor peptide.

To determine the growth inhibitory effect of rAGAP, the cell cycle distribution of SW480 cells was assessed by flow cytometry. In the control group, the proportion of G0/G1 phase cells was 66.5%. Treatment for SW480 cells with 5, 10 and 20 *μ*M rAGAP for 24 h resulted in a significantly increased proportion of G1 phase cells of 83.42, 81.79 and 81.34%, respectively [F(3,8)=23.7, P<0.01]. However, there was no difference in the three drug treatment groups (LSD-t, P<0.05). Meanwhile, rAGAP treatment significantly decreased the proportion of S phase SW480 cells (24.25% of control, 5.47, 7.75 and 7.42% of 5, 10 and 20 *μ*M rAGAP groups, respectively) [F(3,8)=44.85, P<0.01]. The percentage of cells in G2/M phase was 10.87, 10.72 and 11.23% in the 5, 10 and 20 *μ*M rAGAP treatment groups, respectively, compared with that of the control group (9.24%) [F(3,8)=0.59, P<0.05]. Moreover, the rate of apoptotic cells was increased by rAGAP (0.79% for control, 1.45, 2.2 and 3.91% for the 5, 10 and 20 *μ*M rAGAP groups, respectively) [F(3,8)=30.03, P<0.01] (Fig. 3). These results demonstrated that rAGAP treatment resulted in cell cycle arrest in G0/G1 phase and increased apoptosis of SW480 cells.

### Expression of proliferation and apoptosis-related protein by rAGAP treatment

**Figure 4.**
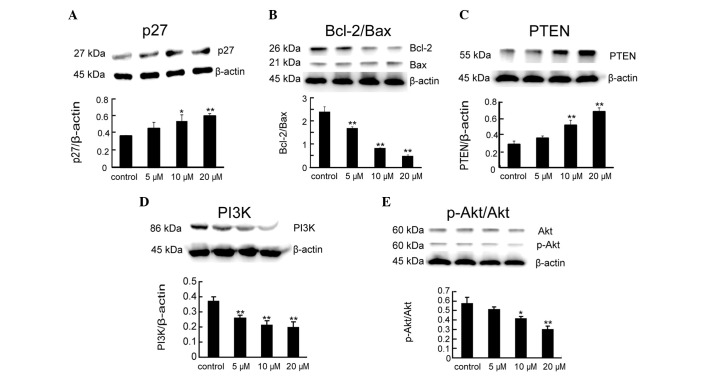
Western blot analysis revealed the changes in the levels of associated proteins in cell cycle inhibition, apoptosis and proliferation of SW480 cells treated with rAGAP. The SW480 cells were treated with rAGAP at 0, 5, 10 and 20 *μ*M. Then the protein was prepared and analyzed as described in Materials and methods. ^*^P<0.05 vs. control, ^**^P<0.01 vs. control, n=3. rAGAP, recombinant analgesic-antitumor peptide.

Compared with the control group, the protein expression of p27, a key effector of cell cycle arrest, was significantly increased by rAGAP treatment for 24 h [F(3,8)=9.6, P<0.05] (Fig. 4A). To explore the mechanism of rAGAP on SW480 cell apoptosis, the levels of Bcl-2 and Bax was measured following rAGAP treatment. The results showed that anti-apoptotic gene Bcl-2 was significantly downregulated. By contrast, the expression of pro-apoptotic gene Bax was upregulated. Thus, the ratios of Bcl-2/Bax in SW480 cells with 24 h treatment of different concentrations of rAGAP was significantly reduced compared to the control group [F(3,8)=145.1, P<0.01] (Fig. 4B).

Since PTEN-PI3K-Akt signaling plays a critical role in cell survival, the expression of PTEN, PI3K and Akt in SW480 cells after 24 h rAGAP treatment were measured by western blot analysis. As shown in Fig. 4C–E, rAGAP increased the levels of PTEN [F(3,8)=46.14, P<0.01] but decreased the levels of PI3K [F(3,8)=73.74, P<0.01] and p-Akt/Akt [F(3,8)=25.19, P<0.01] compared with the control group. However, the expression of total Akt did not impact significantly on the protein levels [F(3,8)=4.02, P<0.05].

## Discussion

Currently, resistance to chemotherapy is becoming a major issue in the long-term treatment of CRC patients. Increasing evidence shows that metastatic CRC patients with v-Ki-ras2 Kirsten rat sarcoma viral oncogene homolog (KRAS) mutations are resistant to treatment with monoclonal antibodies such as cetuximab and panitumumab that target the epidermal growth factor receptor (7,8). Notably, a number of chemotherapy drugs currently in use are natural products or derived directly from natural sources. Therefore, it is feasible to explore natural products as a potential choice for novel anti-cancer therapeutics (9,10).

As a traditional Chinese medicine, Quan Xie (the scorpion *Buthus martensii* Karsch) has been used in the treatment of numerous diseases, including tetanus, tuberculosis, apoplexy, epilepsy, spasm and migraine. Scorpion venom is a complex mixture of low molecular weight bioactive molecules, small peptides and enzymes (11,12). Various different toxic peptides extracted from scorpion venom have different functions. *Buthus martensii* Karsch chlorotoxin-like toxin (BmKCT) has a similar function to CTX, which inhibits the growth and metastasis of glioma cells (13). Indian black scorpion (*Heterometrus bengalensis*) venom inhibits the growth of leukemic U937 and K562 cells by inducing cell apoptosis. Flow cytometric assay revealed that *Heterometrus bengalensis* venom blocked the cell cycle at sub-G1 phase (14). BMK-CBP, a serine proteinase-like protein isolated from the venom of Chinese scorpion (*Buthus martensii* Karsch) dose-dependently inhibits the proliferation of the MCF-7 cancer cell line (15). AGAP is a peptide extracted from scorpion venom. Previous studies showed that AGAP inhibited the mRNA transcription of Nav1.5. Therefore, it is suggested that AGAP may be a Na^+^-channel specific inhibitor (4). rAGAP, a fusion protein consisting of a hexa-histidine (His6) tag, SUMO and AGAP, was overexpressed in *E. coli*(5). The antitumor activity of rAGAP has been confirmed in several studies (4,5).

The cell cycle consists of the pre-DNA synthesis phase (G1), DNA synthesis phase (S), DNA post-synthetic phase (G2) and the phase of mitosis (M). In addition, cells are able to stop mitosis and move from G1 phase of the cell cycle into G0 phase (stationary phase) temporarily. Cell cycle arrest is one of the most important mechanisms of antitumor drug action. If the cell cycle is blocked in G1 phase, unlimited proliferation of tumor cells would be controlled (16,17). Our MTT experimental results indicated the notable ability of rAGAP to inhibit the variability of SW480 cells. Moreover, flow cytometry assay showed that rAGAP induced SW480 cell cycle arrest at G0/G1 phase, accompanied by the reduction in S phase but no significant change in G2/M phase. As a result, the cell cycle could not pass through the G1/S restriction point and was prevented from G1 to S phase transition (18,19).

To determine the molecular mechanism of G0/G1 cell cycle arrest induced by rAGAP, we further examined the expression of cell cycle regulatory protein p27 and the PTEN/PI3K/Akt pathway. p27, a member of the CDK inhibitor family, is a tumor suppressor gene which blocks phosphorylation of the Rb protein, thus inhibiting cell growth and proliferation. When growth signals are lacking, p27 accumulates in cells and combines with cyclin E-CDK2 to inhibit its function. Thereby, p27 is a potential mediator of extracellular stimulation signals to regulate the cell cycle and negatively regulates the cell cycle to inhibit cell growth and tumor formation. p27 protein stability can also be regulated by the PTEN/PI3K/Akt pathway (20–22). The PI3K pathway, which is activated in G1/S transition, contributes to cell proliferation, growth and resistance to therapy for a number of cancers. An accumulation of evidence supports a key role for the PI3K pathway in cell cycle progression (23,24). PTEN is the only tumor suppressor gene involved in the PI3K/Akt pathway. The PTEN/PI3K/Akt pathway not only inhibits G1/S cell cycle progression but also plays a key role in G2/M transition and its constitutive activation may lead to defects in DNA damage checkpoint control (25). Our results showed that rAGAP upregulated the expression of p27 and PTEN, while it downregulated PI3K and p-Akt expression. Therefore, we proposed that rAGAP was capable of increasing the amount of p27 protein and inhibiting PI3K/Akt signal transduction, subsequently leading to G0/G1 cell cycle arrest in SW480 cells.

Apoptosis is an active, programmed and self-destructive process of gene regulation. In general, the initiation of apoptosis is divided into intrinsic and extrinsic apoptotic pathways, which differ in the activation of the signaling pathways that lead to death. Mitochondria and cell surface receptors mediate the two main pathways of apoptosis. In the current study, the intrinsic apoptosis pathway involved with mitochondria was examined by DAPI and flow cytometry, and the results indicated that rAGAP had some induction effect on apoptosis. Expression of cell apoptosis related proteins such as Bcl-2 and Bax as well as PTEN/PI3K/Akt pathway were examined to explore the molecular mechanism of apoptosis induction. It is reported that Bcl-2 family proteins regulate mitochondrial membrane permeability and play an important role in the mitochondrial apoptosis pathway (6). Bcl-2 family proteins includes anti-apoptotic protein (Bcl-xl, Bcl-2, KSHV-Bcl-2 and Bcl-w) and pro-apoptotic proteins (Bax, Bad and Bid) (26). The Bcl-2/Bax ratio may play a key role in deciding whether the cell switches towards proliferation or apoptosis (27,28). It has been reported that the PTEN/PI3K/Akt pathway is constitutively activated in several types of cancer (29). The PTEN/PI3K/Akt pathway is also involved in cell apoptosis progression. PTEN plays a significant role not only in inducing cell cycle arrest but also programming apoptosis (30). Loss of PTEN expression is present in 20–40% of CRC tumors assessed by immunohistochemistry (31–33). PI3K is a lipid kinase that plays an important role in cell growth, survival and resistance to therapy in a number of different solid and liquid cancers. Furthermore, Akt is able to suppress cell apoptosis by inhibiting Bax activation (34,35). Once activated, p-Akt phosphorylates other proteins such as NFκB, mTOR, Bad, GSK-3β and MDM-2 to enhance cell growth, metabolism, survival and proliferation (36–38). Certain studies suggest that second mitochondria-derived activator of caspase (Smac) release is suppressed by Akt, Bcl-2 and Bcl-xl, but promoted by Bax, Bad, and Bid (34,35,39,40). This evidence suggests that PTEN/PI3K/Akt is not only involved in the regulation of the apoptotic process directly but also has interaction with Bcl-2 family members. Our study revealed that rAGAP upregulated the expression of Bax and caused simultaneous downregulation of Bcl-2, thereby decreasing the Bcl-2/Bax ratio. The decrease of Bcl-2 protein level and binding by nonphosphorylated Bad caused the release of Bax, which was translocated into the mitochondria, where it activated the mitochondrial apoptosis pathway (41). As a result, reduction of the ratio of Bcl-2/Bax subsequently enhanced cell apoptosis. Moreover, our study showed that rAGAP significantly increased expression of PTEN, and decreased expression of PI3K and phosphorylation activation of Akt. Therefore, the intrinsic apoptotic pathway (regulating the Bcl-2 family) and the PTEN/PI3K/Akt pathway were involved in rAGAP-induced SW480 colon cancer cell apoptosis.

Taken together, rAGAP inhibits proliferation and induces apoptosis of SW480 human colon cancer cells. The antitumor mechanism of rAGAP may contribute to the increase in p27 expression, reduced ratio of Bcl-2/Bax and inhibition of PTEN/PI3K/Akt signal transition. Our results suggest that rAGAP has the potential to be developed into new therapeutic agents for CRC.

## References

[1] Center MM, Jemal A, Smith RA, Ward E (2009). Worldwide variations in colorectal cancer. CA Cancer J Clin.

[2] Ferlay J, Shin HR, Bray F, Forman D, Mathers C, Parkin DM (2010). Estimates of worldwide burden of cancer in 2008: GLOBOCAN 2008. Int J Cancer.

[3] Weekes J, Lam AK, Sebesan S, Ho YH (2009). Irinotecan therapy and molecular targets in colorectal cancer: a systemic review. World J Gastroenterol.

[4] Zhao Y, Cai X, Ye T (2011). Analgesic-antitumor peptide inhibits proliferation and migration of SHG-44 human malignant glioma cells. J Cell Biochem.

[5] Cao P, Yu J, Lu W (2010). Expression and purification of an antitumor-analgesic peptide from the venom of Mesobuthus martensii Karsch by small ubiquitin-related modifier fusion in *Escherichia coli*. Biotechnol Prog.

[6] Guo Y, Srinivasula SM, Druilhe A, Fernandes-Alnemri T, Alnemri ES (2002). Caspase-2 induces apoptosis by releasing proapoptotic proteins from mitochondria. J Biol Chem.

[7] Amado RG, Wolf M, Peeters M (2008). Wild-type KRAS is required for panitumumab efficacy in patients with metastatic colorectal cancer. J Clin Oncol.

[8] Karapetis CS, Khambata-Ford S, Jonker DJ (2008). K-ras mutations and benefit from cetuximab in advanced colorectal cancer. N Engl J Med.

[9] Deorukhkar A, Krishnan S, Sethi G, Aggarwal BB (2007). Back to basics: how natural products can provide the basis for new therapeutics. Expert Opin Investig Drugs.

[10] Newman DJ, Cragg GM (2007). Natural products as sources of new drugs over the last 25 years. J Nat Prod.

[11] du Plessis LH, Elgar D, du Plessis JL (2008). Southern African scorpion toxins: an overview. Toxicon.

[12] Rodriguez de la Vega RC, Possani LD (2005). Overview of scorpion toxins specific for Na+ channels and related peptides: biodiversity, structure-function relationships and evolution. Toxicon.

[13] Fan S, Sun Z, Jiang D (2010). BmKCT toxin inhibits glioma proliferation and tumor metastasis. Cancer Lett.

[14] Das Gupta S, Debnath A, Saha A (2007). Indian black scorpion (*Heterometrus bengalensis* Koch) venom induced antiproliferative and apoptogenic activity against human leukemic cell lines U937 and K562. Leukemia.

[15] Gao R, Zhang Y, Gopalakrishnakone P (2008). Purification and N-terminal sequence of a serine proteinase-like protein (BMK-CBP) from the venom of the Chinese scorpion (*Buthus martensii* Karsch). Toxicon.

[16] Chaudhry MA (2007). Base excision repair of ionizing radiation-induced DNA damage in G1 and G2 cell cycle phases. Cancer Cell Int.

[17] Sitko JC, Yeh B, Kim M (2008). SOCS3 regulates p21 expression and cell cycle arrest in response to DNA damage. Cell Signal.

[18] Narbonne-Reveau K, Lilly M (2009). The Cyclin-dependent kinase inhibitor Dacapo promotes genomic stability during premeiotic S phase. Mol Biol Cell.

[19] Liu S, Yamauchi H (2009). p27-Associated G1 arrest induced by hinokitiol in human malignant melanoma cells is mediated via down-regulation of pRb, Skp2 ubiquitin ligase, and impairment of Cdk2 function. Cancer Lett.

[20] Connor MK, Kotchetkov R, Cariou S (2003). CRM1/Ran-mediated nuclear export of p27(Kip1) involves a nuclear export signal and links p27 export and proteolysis. Mol Biol Cell.

[21] Yakes FM, Chinratanalab W, Ritter CA, King W, Seelig S, Arteaga CL (2002). Herceptin-induced inhibition of phosphatidylinositol-3 kinase and Akt is required for antibody-mediated effects on p27, cyclin D1, and antitumor action. Cancer Res.

[22] Kerkhoff E, Simpson JC, Leberfinger CB (2001). The Spir actin organizers are involved in vesicle transport processes. Curr Biol.

[23] Jones SM, Kazlauskas A (2001). Growth-factor-dependent mitogenesis requires two distinct phases of signalling. Nat Cell Biol.

[24] Liang J, Zubovitz J, Petrocelli T (2002). PKB/Akt phosphorylates p27, impairs nuclear import of p27 and opposes p27-mediated G1 arrest. Nat Med.

[25] Liang J, Slingerland JM (2003). Multiple roles of the PI3K/PKB (Akt) pathway in cell cycle progression. Cell Cycle.

[26] Petros AM, Olejniczak ET, Fesik SW (2004). Structural biology of the Bcl-2 family of proteins. Biochim Biophys Acta.

[27] Chowdhury I, Tharakan B, Bhat GK (2008). Caspases - an update. Comp Biochem Physiol B Biochem Mol Biol.

[28] D'Amelio M, Tino E, Cecconi F (2008). The apoptosome: emerging insights and new potential targets for drug design. Pharm Res.

[29] Vivanco I, Sawyers CL (2002). The phosphatidylinositol 3-Kinase AKT pathway in human cancer. Nat Rev Cancer.

[30] Di Cristofano A, Pandolfi PP (2000). The multiple roles of PTEN in tumor suppression. Cell.

[31] Laurent-Puig P, Cayre A, Manceau G (2009). Analysis of PTEN, BRAF, and EGFR status in determining benefit from cetuximab therapy in wild-type KRAS metastatic colon cancer. J Clin Oncol.

[32] Loupakis F, Pollina L, Stasi I (2009). PTEN expression and KRAS mutations on primary tumors and metastases in the prediction of benefit from cetuximab plus irinotecan for patients with metastatic colorectal cancer. J Clin Oncol.

[33] Frattini M, Saletti P, Romagnani E (2007). PTEN loss of expression predicts cetuximab efficacy in metastatic colorectal cancer patients. Br J Cancer.

[34] Vyas S, Juin P, Hancock D (2004). Differentiation-dependent sensitivity to apoptogenic factors in PC12 cells. J Biol Chem.

[35] Majewski N, Nogueira V, Robey RB, Hay N (2004). Akt inhibits apoptosis downstream of BID cleavage via a glucose-dependent mechanism involving mitochondrial hexokinases. Mol Cell Biol.

[36] Engelman JA, Luo J, Cantley LC (2006). The evolution of phosphatidylinositol 3-kinases as regulators of growth and metabolism. Nat Rev Genet.

[37] Carnero A (2010). The PKB/AKT pathway in cancer. Curr Pharm Des.

[38] Song G, Ouyang G, Bao S (2005). The activation of Akt/PKB signaling pathway and cell survival. J Cell Mol Med.

[39] Liu J, Yin S, Reddy N, Spencer C, Sheng S (2004). Bax mediates the apoptosis-sensitizing effect of maspin. Cancer Res.

[40] Maianski NA, Geissler J, Srinivasula SM, Alnemri ES, Roos D, Kuijpers TW (2004). Functional characterization of mitochondria in neutrophils: a role restricted to apoptosis. Cell Death Differ.

[41] Kang MH, Reynolds CP (2009). Bcl-2 inhibitors: targeting mitochondrial apoptotic pathways in cancer therapy. Clin Cancer Res.

